# Epidemiology of pediatric meningitis and encephalitis in Japan: a cross-sectional study

**DOI:** 10.1128/spectrum.01192-24

**Published:** 2024-10-04

**Authors:** Shinsuke Mizuno, Yoshiki Kusama, Shogo Otake, Yusuke Ito, Masatoshi Nozaki, Masashi Kasai

**Affiliations:** 1 Division of Infectious Diseases, Department of Pediatrics, HyogoPrefectural Kobe Children’s Hospital, Kobe City, Hyogo, Japan; 2 Division of Infectious Control and Prevention, Graduate School of Medicine/Faculty of Medicine, Osaka University, Osaka, Japan; 3 Department of Pediatrics, Kobe University Graduate School of Medicine, Hyogo, Japan; 4 Division of Pediatric Infectious Diseases, Department of Pediatrics, HyogoPrefectural Amagasaki General Medical Center, Hyogo, Japan; 5 Department of Perinatal and Pediatrics Infectious Diseases, Osaka Women’s and Children’s Hospital, Izumi City, Osaka, Japan; City of Hope Department of Pathology, Duarte, California, USA

**Keywords:** meningitis, encephalitis, children, epidemiology, human parechovirus, enterovirus, bacterial meningitis

## Abstract

Information on the epidemiology and cause-specific clinical features of pediatric meningitis and encephalitis is limited, owing to conventional laboratory methods' limitations in identifying causative pathogens. The FilmArray Meningitis/Encephalitis (FA-M/E) panel, a molecular diagnostic tool, can detect 14 pathogens within 1 hour. We investigated meningitis and encephalitis epidemiology among children in Japan and FA-M/E panels' utility in their clinical management. This cross-sectional study was conducted among children aged 0–18 years admitted to seven regional hospitals in western Japan between October 2022 and September 2023. Cerebrospinal fluid (CSF) samples were collected from 221 children and tested using the FA-M/E panel. Clinical and microbiological data were reviewed retrospectively. Fifty-eight patients tested positive using the FA-M/E panel. Viral and bacterial pathogens were detected in 49 and nine cases, respectively. Human parechovirus and enterovirus occurred mainly in epidemic clusters during the summer and were primarily detected in young infants. Patients who tested positive for human parechovirus had a significantly higher frequency of sepsis-like manifestations and a lower frequency of CSF pleocytosis than did those who tested positive for enterovirus. CSF pleocytosis was absent in 30 patients who tested positive using the FA-M/E panel. Among the patients who tested positive for bacteria, three of the nine were not diagnosed using conventional culture methods, owing to prior antimicrobial therapy. The FA-M/E panel can identify bacterial and viral pathogens causing pediatric meningitis and characterize the epidemiology in local communities.

**IMPORTANCE:**

Culture and polymerase chain reactions have traditionally been used to identify microorganisms causing pediatric meningitis and encephalitis. However, the methods currently used to identify the causative microorganisms are limited, particularly in general hospitals. The FilmArray Meningitis/Encephalitis (FA-M/E) panel, a fully automated genetic testing system, can detect 14 pathogens using the multiplex polymerase chain reaction method. This study described the epidemiology of pediatric meningitis and encephalitis in Japan. The microorganisms causing acute meningitis and encephalitis in children in Japan were identified using the FilmArray Meningitis/Encephalitis panel. Testing cerebrospinal fluid using the FA-M/E panel is useful for the identification of the pathogen in children with community-acquired acute meningitis and encephalitis. This increases knowledge on the epidemiology and clinical manifestations of acute meningitis and encephalitis caused by specific pathogens and can be used to facilitate optimal patient management.

## INTRODUCTION

Culture and polymerase chain reaction (PCR) have traditionally been used to identify microorganisms causing pediatric meningitis and encephalitis. In Japan, cases of bacterial meningitis and aseptic meningitis must be reported by designated sentinel medical facilities in accordance with the Act on the Prevention of Infectious Diseases and Medical Care for Patients with Infectious Diseases (1). However, the methods currently used to identify the causative microorganisms are limited, particularly in general hospitals. Consequently, the pathogen is not identified in many cases, and information on the epidemiology of meningitis and encephalitis in children according to the causative microorganism is limited. A nationwide survey conducted in Japan between 2019 and 2021 by Furuichi et al. (2) identified pathogens in 125 cases of pediatric bacterial meningitis. Group B *Streptococcus* (GBS), *Streptococcus pneumoniae*, *Escherichia coli*, and *Listeria monocytogenes* were the most commonly detected bacterial pathogens (48%, 21%, 10%, and 5%, respectively) in this survey. Among 925 patients with aseptic meningitis identified by National Sentinel Surveillance conducted between 2020 and 2021, 216 patients underwent further testing at local public health institutes to identify the causative microorganisms (3). Enterovirus (EV) was identified as the cause of aseptic meningitis in the majority of these patients, accounting for 79.6% (172/216) of cases.

In recent years, nucleic acid amplification methods such as PCR have gained popularity in clinical practice for confirming the presence of specific pathogens. The FilmArray Meningitis/Encephalitis (FA-M/E) panel (BioFire Diagnostics, LLC, Salt Lake City, UT, USA), a fully automated genetic testing system, can detect 14 pathogens, namely *E. coli* (K1 capsular type), *Haemophilus influenzae*, *L. monocytogenes*, *Neisseria meningitidis*, *Streptococcus agalactiae*, *Streptococcus pneumoniae*, EV, herpes simplex virus types 1 and 2 (HSV-1/2), human herpes virus 6 (HHV-6), human parechovirus (HPeV), varicella zoster virus, cytomegalovirus, and *Cryptococcus neoformans* or *gattii* in the cerebrospinal fluid (CSF) (4). This system can rapidly and accurately detect nucleic acid of multiple pathogens using the multiplex PCR method. Moreover, it requires only 200 µL of CSF per sample and provides results in approximately 1 hour. The FA-M/E panel received medical device marketing approval in Japan in April 2019 and has been covered by insurance since October 2022. Previous studies have demonstrated the accuracy and clinical utility of FA-M/E in adults from North America and some European countries (5–8). However, current data regarding the use of FA-M/E in children in the Asia-Pacific region, with supporting epidemiological and clinical data, are lacking. Therefore, this study aimed to investigate the epidemiology of meningitis and encephalitis among children aged 0–18 years and evaluate the utility of the FA-M/E panel in their clinical management in Japan.

## MATERIALS AND METHODS

### Study design and setting

**Fig 1:**
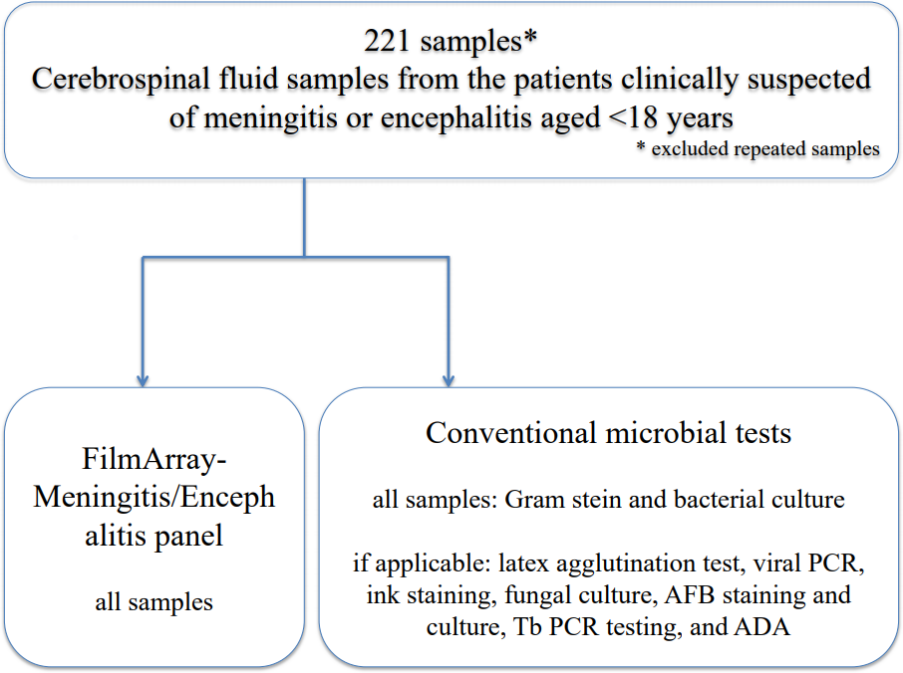
The algorithm for conventional microbial tests and the FA-M/E panel.

Pediatric patients aged 0–18 years who visited five tertiary pediatric care hospitals and two specialized pediatric hospitals in the western region of the main island in Japan between 01 October 2022 and 30 September 2023 were eligible for inclusion in this multi-center cross-sectional study. The algorithm for conventional microbial tests and the FA-M/E panel is shown in Fig. 1. The FA-M/E panel was done for samples clinically suspected of meningitis or encephalitis. Patients who had provided CSF samples for analysis with the FA-M/E panel were included in the analysis. Repeated samples from the same patient were excluded. FA-M/E was performed as a routine examination in the bacteriology laboratories of each medical institution in accordance with the instructions provided in the manual. The FA-M/E panel consists of automated nucleic acid extraction, reverse transcription, and nucleic acid amplification. A total of 200 µL of uncentrifuged CSF and hydration solution was drawn into the FA-M/E reagent pouch and then placed in a FilmArray (version 2.0) instrument and tested. CSF samples were stored under refrigeration (4°C) for up to 7 days. The results were immediately reported to the attending physicians on the same day as the sample collection, and the physician interpreted them using clinical information.

### Conventional methods

Conventional methods comprise CSF Gram staining, as well as bacterial culture for CSF and blood, which were performed on all samples. If available at the facility, the latex agglutination test was conducted. Additionally, viral DNA PCR test for herpes simplex virus (HSV), ink staining, and fungal culture, acid-fast bacterial staining, PCR testing, and adenosine deaminase measurement were conducted in cases clinically suspected of specific infections. Standard culture media were used for culture, and standard biochemical and phenotype analyses were performed to identify the bacterial and fungal pathogens isolated.

### Data collection

Clinical data, comprising anonymized information, such as age, sex, blood test results, CSF test results (including the FA-M/E panel), administration of antimicrobial and anti-herpetic drugs before lumber puncture, brain imaging findings, and patient outcomes, were extracted from the electronic medical records.

### Definitions

The clinical signs of meningitis include nuchal rigidity, photophobia, paradoxical irritability, and bulging fontanel. *Sepsis* was defined as suspected or confirmed infection and the presence of systemic inflammatory response syndrome (9). *Systemic inflammatory response syndrome* was defined as meeting more than two of the following criteria, one of which one must be an abnormal body temperature or white blood cell (WBC) count: (1) pyrexia or hypothermia, (2) age-dependent tachycardia or bradycardia, (3) tachypnea or the need for mechanical ventilation, or an abnormal WBC count or >10% immature neutrophils (9). *Pleocytosis*, characterized by an increase in the WBC count in the CSF, was defined as ≥15 WBCs/µL in neonates, ≥10 WBCs/µL in infants aged 1–3 months, and ≥9 or more WBCs/µL in children aged ≥3 months. Abnormal glucose levels in the CSF were defined as >25 mg/dL in neonates, >27 mg/dL in infants aged 1–3 months, and >34 mg/dL in children aged ≥3 months. Similarly, protein levels exceeding >127 mg/dL in neonates, 100 mg/dL in infants aged 1–3 months, and 170 mg/dL in children aged ≥3 months were considered abnormal (10).

### Statistical methods

Descriptive analyses were performed on clinical and outcome data, including clinical presentation on admission, laboratory data, and final diagnosis at discharge. Categorical variables are expressed as count (%), and continuous variables are expressed as the median and interquartile range (IQR). Comparisons between the two groups were performed using Fisher's exact test for categorical variables. Statistical significance was set at *P* < 0.05. Statistical analyses were performed using IBM SPSS Statistics for Windows, version 27 (IBMS Corp., Armonk, NY, USA).

## RESULTS

### Demographics characteristics and summary of the FA-M/E panel findings

A total of 221 CSF specimens were tested using the FA-M/E panel during the study period. The patients' background characteristics are shown in Table 1. The age at the onset of disease was as follows: <1 month, 22.2% (49/221); 1–3 months, 25.3% (56/221); 3–24 months, 19.5% (43/221); and >24 months, 33.0% (73/221). Moreover, 41.6% (92/221) of the enrolled patients were girls. Fever, headache (if the patient could express it appropriately), and vomiting were present in 81.9% (181/221), 7.7% (17/221), and 15.4% (34/221) of patients, respectively.

**TABLE 1:** Patient's background characteristics

Variables	Number	Proportion (%)
Age (number (%))		
<1 month	49	22.2
1 to 3 months	56	25.3
3 to 24 months	43	19.5
>24 months	73	33.0
Sex (girl number, %)	92	41.6
Fever (number, %)	181	81.9
Neurological symptoms (number, %)		
Headache	17	7.7
Vomit	34	15.4
Altered mental status	43	19.5
Abnormal neurological examination	32	14.5
Seizure	74	33.5
Cerebrospinal fluid findings		
Pleocytosis	80	36.2
High protein level	23	10.4
Low glucose level	6	2.7
Outcome		
Recovery	182	82.4
Neurological sequelae	30	13.6
Died	2	0.9
Other	7	3.2

**Fig 2:**
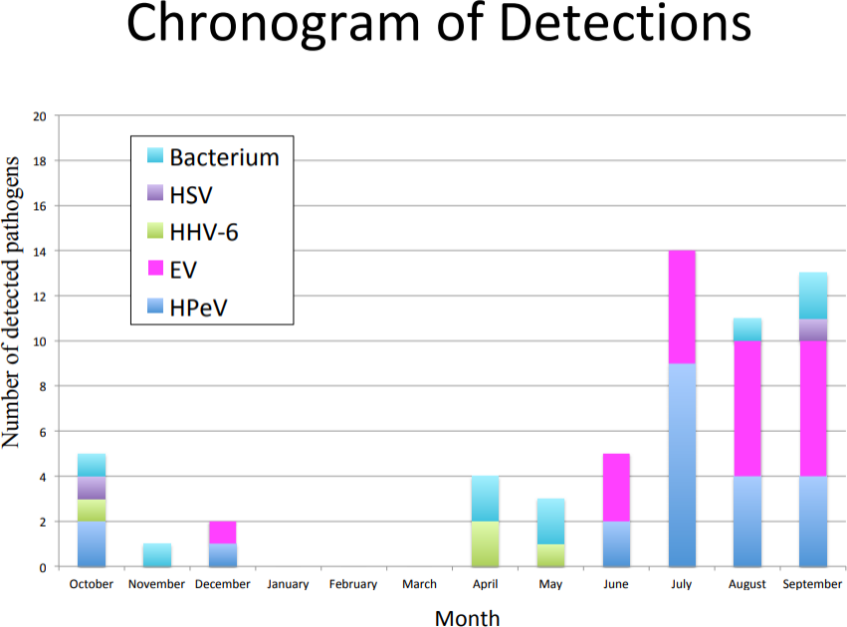
The type of microorganisms detected by month.

Pathogenic microorganisms were identified in 58 (26.2%) of the 221 cases using the FA-M/E panel, and two of the six patients who tested positive for HHV-6 were excluded from further evaluation because they had no clinically relevant findings (Table 2). HPeV was the most frequently detected pathogen (22 cases), followed by EV (21 cases). Bacterial pathogens were detected among the nine patients via the FA-M/E panel (GBS in four cases, *S. pneumoniae* in four cases, and *E. coli* in one case). Figure 2 shows the type of pathogen detected by month. Most HPeV and EV were detected between July and September; however, other pathogens were detected throughout the year.

**TABLE 2:** Identified microorganisms using FilmArray Meningitis/Encephalitis panel

Microorganism	Number	Proportion (%)
Positive result	58	26.2
Virus	49	22.2
*Human parechovirus*	22	10.0
*Enterovirus*	21	9.5
*Human herpesvirus-6*	4	1.8
*Human herpesvirus-2*	2	0.9
Bacterium	9	4.1
*Streptococcus agalactiae*	4	1.8
*Streptococcus pneumoniae*	4	1.8
*Escherichia coli*	1	0.5

Table 3 shows the final diagnosis of the patients at the time of discharge. The FA-M/E panel did not detect any pathogens in 163 cases (73.8%). The most common final diagnosis was viral meningitis or encephalitis. In young infants with negative FA-M/E panel results, the most common diagnoses other than viral meningitis or encephalitis were fever (42 cases), febrile seizure with unknown etiology (24 cases), acute encephalopathy (23 cases), and epilepsy (18 cases). Coagulase-negative *Staphylococcus* (two patients with ventricular-perinatal shunt-related infection), *Serratia marcescens* (one neonate with meningitis), and *Streptococcus gallolyticus* (one neonate with meningitis) were detected in four bacterial cultures (Supplementary Material). The causative organism was not identified in eight cases of aseptic meningitis. Four patients were diagnosed with autoimmune encephalitis and received immunotherapy.

**TABLE 3:** The final diagnosis of the patients at the time of discharge

	FA-M/E positive (*N* = 58)	FA-M/E negative (*N* = 163)
Viral meningitis/encephalitis	49	8
Febrile infant with/without focus	0	42
Febrile seizure with unknown etiology^*a*^	0	24
Acute encephalopathy	0	23
Epileptic seizure	0	18
Bacterial meningitis/encephalitis	9	4
Apnea	0	7
Drug-induced encephalopathy	0	5
Trauma	0	5
Autoimmune encephalitis	0	4
Cerebrovascular diseases	0	4
Somatoform disorder	0	4
Others^*b*^	0	15

^*a*^ Febrile infant: acute respiratory infection (12), febrile urinary tract infection (11), bacteremia (8), sepsis (4), without any sources (7).

^*b*^ Others: congenital cytomegalovirus infection (2), hypoglycemia (2), Kawasaki disease (2), transverse myelitis (2), central nervous system lymphoma (1), infected cephalohematoma (1), myasthenia gravis (1), orbital cellulitis (1), retropharyngeal abscess (1), delirium (1), and neonatal herpes simplex virus infection limited to the skin, eye and mouth (1); FA-M/E, FilmArray Meningitis/Encephalitis panel.

### Clinical characteristics, laboratory findings, and outcomes of the patients with FA-M/E-positive results

Table 4 presents the clinical characteristics of patients with FA-M/E-positive results. Both HPeV and EV were detected primarily in neonates and young infants during the summer of 2023, suggesting the presence of epidemics in the western region of the main island in Japan (Fig. 1) during this period. Contact of the patients with family members with infection was reported in 15 of the 22 cases caused by HPeV and 14 of the 21 cases caused by EV. Twenty-one of the 22 cases were caused by HPeV and all 21 cases were caused by EV presented with fever. Signs of sepsis were more common in patients with HPeV (11 of 22 cases) than in those with EV (three of 21 cases) (*P* = 0·022). The incidence of meningeal signs, abnormal findings on neurological examination, and seizures were similar between the patients with HPeV and EV. Pleocytosis in CSF was less common in the patients with HPeV (three of 22 cases) than in those with EV (14 of 21 cases) (*P* = 0·001).

**TABLE 4:** The clinical characteristics of the patients with FA-M/E-positive results^*a*^

Variables	Virus				Bacterium			Total
	HPeV	EV	HHV6	HSV2	GBS	SP	EC	
Case number	22	21	4	2	4	4	1	58
Age, number (%)								
<1 month	6 (27.3)	11 (52.4)	0	0	2 (50)	0	1 (100)	20
1 to 3 months	16 (72.7)	7 (33.3)	1 (25)	0	2 (50)	0	0	26
3 to 24 months	0	0	2 (50)	2 (100)	0	3 (75)	0	7
>24 months	0	3 (14.3)	1 (25)	0	0	1 (25)	0	5
Sick contact, number (%)								
Parents	3 (13.6)	5 (23.8)	1 (25)	1 (50)	0	0	0	10
Siblings	12 (54.5)	9 (42.9)	0	0	0	0	0	21
Others	0	0	0	0	0	0	0	0
No	7 (31.8)	7 (33.3)	3 (75)	1 (50)	4 (100)	4 (100)	1 (100)	27
Symptoms, number (%)								
Fever	21 (95.5)	21 (100)	3 (75)	1 (50)	3 (75)	4 (100)	0	53
Sepsis-like symptoms	11 (50)	3 (14.3)	0	0	1 (25)	0	0	15
Meningeal signs	6 (3, 27)	5 (23.8)	4 (100)	0	0	3 (75)	0	18
Abnormal neurological findings	9 (40.9)	6 (28.6)	4 (100)	0	0	3 (75)	0	22
Seizure	1 (4.5)	4 (19.0)	3 (75)	2 (100)	1 (25)	0	0	11
Timing of LP from onset, number (%)								
<24 h	14 (63.6)	12 (57.1)	1 (25)	0	3 (75)	0	1 (100)	31
24–72 h	7 (31.8)	3 (14.3)	2 (50)	0	1 (25)	1 (25)	0	16
>72 h	1 (4.5)	2 (9.5)	1 (25)	2 (100)	0	3 (75)	0	11
ABx administration prior to LP, number (%)								
Yes	7 (31.8)	2 (9.5)	1 (25)	0	2 (50)	1 (25)	1 (100)	14
Blood test findings, median								
WBC × 10^3^ /µL	6.2	11.9	6.4	9.2	6.0	20.2	22.1	7.8
CRP mg/dL	0.20	0.18	0.22	0.05	2.38	20.81	3.1	0.24
CSF test findings, number (%)								
Pleocytosis	3 (13.6)	14 (66.7)	1 (25)	2 (100)	4 (100)	4 (100)	0	28
Elevated TP	2 (9.1)	1 (4.8)	0	0	4 (100)	1 (25)	0	8
Decreased glucose	0	0	0	0	1 (25)	3 (75)	0	4
Brain imaging test findings, number (%)								
No image	13 (59.1)	12 (57.1)	1 (25)	0	0	0	0	26
Abnormal/total image	5/9	4/9	2/3	1/2	4/4	2/4	0/1	18/32
Prognosis, number (%)								
Neurological sequelae	0	2 (9.5)	1 (25)	0	1 (25)	1 (25)	0	5
Death	0	0	0	0	0	0	0	0

^*a*^ ABx, antibiotics; CRP, C-reactive protein; CSF, cerebrospinal fluid; EC, *Escherichia coli*; EV, enterovirus; FA-M/E, FilmArray Meningitis/Encephalitis panel; GBS, Group B *Streptococcus*; HHV-6, human herpes virus 6; HPeV, human parechovirus; HSV, herpes simplex virus; SP, *Streptococcus pneumoniae*; TP, total protein; WBC, white blood cell.

Most patients with HPeV and EV infections had normal CSF protein and glucose levels, normal WBC counts, and normal C-reactive protein levels. Imaging test results were available for nine of the 22 HPeV-positive patients, of whom five (56%) exhibited abnormal findings. Similarly, results were available for nine of the 21 EV-positive patients, of whom four (44%) presented with abnormal findings. None of the 22 patients with HPeV and two of the 21 patients with EV had neurological sequelae at discharge. None of the patients with HPeV or EV infection died prior to discharge.

The clinical characteristics of the other pathogens, including four cases of HHV-6, two cases of HSV-2, four cases of GBS, four cases of *S. pneumoniae*, and one case of *E. coli*, are also reported in Table 4. Of the patients who tested positive for HSV-2, GBS, and *S. pneumoniae*, one patient each showed neurological sequelae; however, none of the patients died prior to discharge.

Three of the patients with bacterial pathogens had negative CSF cultures. Notably, two of the three patients with negative CSF cultures had received antibiotics prior to CSF sampling. All patients positive for GBS and *E. coli* were neonates or young infants, whereas *S. pneumoniae* was detected in older age groups. All patients who tested positive for GBS and *S. pneumoniae* had CSF pleocytosis, whereas the patients who tested positive for *E. coli* had normal CSF cell counts. Of the nine patients who tested positive for bacterial pathogens, five had elevated CSF protein levels, four had low glucose levels, and seven had abnormal brain imaging findings.

## DISCUSSION

In this study, the FA-M/E panel identified a causative microorganism in approximately 25% of patients tested. The findings of this study confirm the epidemiological importance of HPeV and EV as causes of early infantile meningitis and encephalitis, particularly during the summer. Children with HPeV infection presented with significantly more signs of sepsis and less CSF pleocytosis than children with EV infection. The study results show that the FA-M/E panel can identify the causative microorganism in most cases of bacterial meningitis with negative cultures, especially in those with prior administration of antimicrobial agents. However, several cases of bacterial meningitis did not have a pathogen detected by the FA-M/E panel. Notably, in many patients, the CSF findings were an inadequate indicator of meningitis and encephalitis.

The FA-M/E panel revealed the presence of community-wide epidemics of HPeV and EV meningitis and encephalitis in the summer of 2023. Previous studies using FA-M/E panels in pediatric patients exhibited pathogen detection rates ranging from 11.1% to 25.5%, with EV being the most commonly detected microorganism, with high frequency during the summer and autumn months in regions with temperate climates (11–14). A high frequency of HPeV detection in CSF, particularly during the summer and autumn, has also been reported previously (15, 16). Previous reports have shown that although EV infections cause relatively stable annual epidemics, HPeV infections cause sporadic epidemics every few years (11–17). However, data for the Asia-Pacific region are lacking. This study showed that HPeV and EV were the most commonly detected pathogens, with a high incidence during the summer. National sentinel surveillance reports from 2022 to 2023 also revealed that an HPeV epidemic occurred in some areas of Japan during the summer (18).

In this study, although children with meningitis and encephalitis caused by HPeV and EV were similar in terms of age (neonates and young infants) and seasonality (summer), their clinical and laboratory findings differed. Previous studies have found that almost all children with HPeV infection were young infants, whereas EV infections were widely distributed across most age groups (11–17). The secondary attack rates among family members have been reported to be lower for HPeV meningitis and encephalitis than or EV (19–21). This study showed that all HPeV infections and the majority of EV infections occurred in young infants, and exposure to a family member with infection was confirmed in approximately 70% of cases of HPeV and EV infection. Sepsis-like syndrome has been reported in 40–80% of HPeV (especially type 3) infections and 10–30% of EV infections in young infants (21–27). The lack of pleocytosis in CSF of patients with HPeV is a characteristic not seen in cases of meningitis and encephalitis caused by other microorganisms. In children aged <3 months, approximately 90% of those with HPeV-positive CSF and >50% of those with EV-positive CSF showed normal CSF cell counts, consistent with previous reports' results (21–25). The higher frequency of signs of sepsis in patients with HPeV compared with those with EV in this study is also consistent with previous reports. The reason for the scarcity of pleocytosis in patients with HPeV infections is unknown. It has been hypothesized that although viral leakage from blood to CSF occurs in infants with HPeV infection, the immune response to HPeV in the CSF is low (28, 29). Given that meningitis and encephalitis caused by HPeV and EV generally have a good prognosis and often resolve with supportive care, it is important to consider the pathogens based on the surrounding epidemic and exposure to infection by family members. The FA-M/E panel results and such information could enable physicians to discontinue antimicrobial agents.

This study confirms the usefulness of the FA-M/E panel for diagnosing bacterial meningitis. The FA-M/E panel can detect bacterial pathogens in cases in which antimicrobial agents were administered prior to blood and CSF culture (6, 13, 30, 31). In this study, one case each of GBS, *S. pneumoniae*, and *E. coli* with a history of prior antimicrobial agents had negative bacterial cultures but were identified by FA-M/E panel. Although a negative FA-M/E panel result does not rule out infectious meningitis and encephalitis, it can help determine the treatment strategy when combined with other clinical information (32). Four patients with autoimmune encephalitis and 23 patients with acute encephalopathy had negative FA-M/E panel results in this study. In some patients, the negative FA-M/E panel results, combined with the clinical course and imaging findings, enabled prompt initiation of immunotherapy.

However, the results of the FA-M/E panel must be interpreted with caution. First, false-positive results can occur, especially for *S. pneumoniae* and *S. agalactiae*. False-positive rates have been reported as high as 17.5% for *S. pneumoniae* and 15.4% for *S. agalactiae*, which may lead to unnecessary administration of antimicrobial agents (5). Second, false-negative results can also occur, and some cases of meningitis and encephalitis are caused by microorganisms not identified using the FA-M/E panel. *E. coli* other than K1 and some other bacteria that can cause bacterial meningitis are not included in the FA-M/E panel; therefore, a negative FA-M/E panel does not rule out bacterial meningitis (4–8). In this study, four cases of bacterial meningitis caused by microorganisms not included in the FA-M/E panel were identified using bacterial culture. Next generation sequencing may be helpful in detecting unknown pathogens for meningitis or encephalitis with a more comprehensive search and detail information, including genome sequence evaluation. However, this method requires special equipment and techniques, a longer time for analysis, and a larger volume of samples (sometimes it is difficult to obtain the appropriate volume for small children). Therefore, it is important to use each test properly. Third, this study confirmed that CSF biomarkers have a low negative predictive value for meningitis and encephalitis in children (6, 11, 33). In this study, >50% of patients with FA-M/E panel-positive specimens, including both bacterial and viral cases, had completely normal CSF findings.

This study has some limitations. First, the FA-M/E panel was used at the discretion of physicians at each medical facility. It is unlikely that the criteria for indication for CSF testing and FA-M/E panel deviated significantly from the guidelines for the management of fever in young infants and meningitis and encephalitis because all institutions participating in this study were core regional hospitals and training and teaching facilities. However, selection bias due to individual and institutional differences in indications for requesting FA-M/E panel testing cannot be ruled out. Thus, further prospective studies with uniform criteria for testing CSF and FA-M/E panels are needed. Second, the study was conducted only 1 year after the COVID-19 pandemic and only in the western region of the main island in Japan. Compared with previous studies using FA-M/E panels, HPeV was identified more frequently, which may have reflected an endemic of HPeV infection in several regions in Japan during the study period (11–14). Therefore, longitudinal studies with multiple centers covering a wide geographic area conducted over a longer period are needed to identify epidemiological trends.

An FA-M/E panel test costs 17,000 yen (approximately 110 to 120 dollars) with insurance in Japan. Conversely, an HSV DNA PCR test costs 4,500 yen, and acyclovir administration costs approximately 500 yen/kg/day for several days until test results are known. Thus, the additional cost of the FA-M/E panel is not significantly different from that of conventional methods alone and may contribute to improved clinical outcomes and cost-effectiveness, such as shorter hospital stays and fewer neurological sequela and deaths due to earlier treatment.

In conclusion, the FA-M/E panel can aid in understanding the epidemiological and clinical data of children with acute community-acquired meningitis and encephalitis. This information can be used to facilitate optimal patient management.

## Data Availability

The data supporting this study are available from the corresponding author on reasonable request.

## SUPPLEMENTAL MATERIAL

Supplemental material spectrum.01192-24-s0001.docx The clinical characteristics of the other microorganisms.

ASM does not own the copyrights to Supplemental Material that may be linked to, or accessed through, an article. The authors have granted ASM a non-exclusive, world-wide license to publish the Supplemental Material files. Please contact the corresponding author directly for reuse.
