# Supplementary material for: Epidemiology of pediatric meningitis and encephalitis in Japan: a cross-sectional study
Source: Microbiol Spectr. 2024 Oct 4;12(11):e01192-24. doi: 10.1128/spectrum.01192-24 (PMC11537098; doi:10.1128/spectrum.01192-24)
Supplement: Supplemental material — The clinical characteristics of the other microorganisms. [file spectrum.01192-24-s0001.docx]

Supplementary materials

| Pathogen | Age | Pre ABx | Manifestations | | Duration from onset to test | Cerebrospinal fluid | | | Brain imaging findings | Blood test | | Outcome |
| --- | --- | --- | --- | --- | --- | --- | --- | --- | --- | --- | --- | --- |
|  |  |  | Non-neurological | Neurological |  | Cell count (/μL) | Total protein (mg/dL) | Glucose (mg/dL) |  | WBC (/μL) | CRP (mg/dL) |  |
| CNS | > 24 months | yes | fever | headache, vomit | 24-72 h | 5037 | 163 | 50 | normal | 13600 | 4.6 | full recovery |
| CNS | > 24 months | no | fever | no | 24-72 h | 25080 | 82 | 45 | normal | 14200 | 13 | neurological sequae |
| SG | < 3 months | yes | fever | vomit | < 24 h | 10132 | 456 | 42 | normal | 19800 | 6.7 | neurological sequae |
| SM | 3 to 23 months | no | fever | vomit | < 24 h | 901 | 164 | 37 | normal | 13600 | 4.6 | full recovery |

ABx, antibiotics; CNS, coagulase-negative staphylococcus; CRP, C-reactive protein; FA-M/E, FilmArray meningitis/encephalitis panel; SG, Streptococcus gallolyticus; SM, Serratia marcescens; SP, Streptococcus pneumoniae; WBC, white blood cell
